# A Bayesian comparative effectiveness trial in action: developing a platform for multisite study adaptive randomization

**DOI:** 10.1186/s13063-016-1544-5

**Published:** 2016-08-31

**Authors:** Alexandra R. Brown, Byron J. Gajewski, Lauren S. Aaronson, Dinesh Pal Mudaranthakam, Suzanne L. Hunt, Scott M. Berry, Melanie Quintana, Mamatha Pasnoor, Mazen M. Dimachkie, Omar Jawdat, Laura Herbelin, Richard J. Barohn

**Affiliations:** 1 Department of Biostatistics, University of Kansas Medical Center, Mail Stop 1026, 3901 Rainbow Blvd, Kansas City, KS 66160 USA; 2 School of Nursing, University of Kansas Medical Center, Kansas City, KS 66160 USA; 3 Berry Consultants, 4301 Westbank Drive, Suite 140, Bldg B, Austin, TX 78746 USA; 4 Department of Neurology, University of Kansas Medical Center, Kansas City, KS 66160 USA

**Keywords:** Bayesian adaptive design, Clinical trial conduct, Data capture, Bayesian randomization, Adaptive randomization, Response-adaptive randomization, REDCap

## Abstract

**Background:**

In the last few decades, the number of trials using Bayesian methods has grown rapidly. Publications prior to 1990 included only three clinical trials that used Bayesian methods, but that number quickly jumped to 19 in the 1990s and to 99 from 2000 to 2012. While this literature provides many examples of Bayesian Adaptive Designs (BAD), none of the papers that are available walks the reader through the detailed process of conducting a BAD. This paper fills that gap by describing the BAD process used for one comparative effectiveness trial (Patient Assisted Intervention for Neuropathy: Comparison of Treatment in Real Life Situations) that can be generalized for use by others. A BAD was chosen with efficiency in mind. Response-adaptive randomization allows the potential for substantially smaller sample sizes, and can provide faster conclusions about which treatment or treatments are most effective. An Internet-based electronic data capture tool, which features a randomization module, facilitated data capture across study sites and an in-house computation software program was developed to implement the response-adaptive randomization.

**Results:**

A process for adapting randomization with minimal interruption to study sites was developed. A new randomization table can be generated quickly and can be seamlessly integrated in the data capture tool with minimal interruption to study sites.

**Conclusion:**

This manuscript is the first to detail the technical process used to evaluate a multisite comparative effectiveness trial using adaptive randomization. An important opportunity for the application of Bayesian trials is in comparative effectiveness trials. The specific case study presented in this paper can be used as a model for conducting future clinical trials using a combination of statistical software and a web-based application.

**Trial registration:**

ClinicalTrials.gov Identifier: NCT02260388, registered on 6 October 2014

## Background

Challenging statistical issues often arise when designing, analyzing, and conducting clinical trials that assess safety and effectiveness of treatments [1]. Bayesian methodology offers much to address these challenges. It is well-suited for flexible adaptation and can lead to more efficient trials with more patients receiving better treatment [2]. With recent advances in technologies and the availability of prior information, Bayesian Adaptive Designs (BAD) are ready for broader applications [3]. Indeed, the number of trials using Bayesian methods has grown rapidly over time. Prior to 1990 only three published clinical trials used Bayesian methods, but that number quickly jumped to 19 in the 1990s and to 99 from 2000 to 2012 [4]. Reflecting this increased interest in using Bayesian methodology, the Patient Centered Outcomes Research Institute (PCORI), a leading funder of comparative effectiveness research in the USA, has adopted specific policies and guidelines encouraging the use of BAD in comparative effectiveness trials [5].

Published reports of studies using BAD, however, generally report on the design and results found, but provide little information on the process of adaptation used that would allow others to replicate the study, a key feature for advancing science and confirming evidence for translation into clinical practice. Lee and Chu conducted an extensive literature review to ascertain how Bayesian methods have been applied in the design, implementation, and analysis of real clinical trials. They found that while most trials (62 %) applied Bayesian methods for testing treatment efficacy, only about 5 % looked at both efficacy and safety, approximately 5 % applied adaptive randomization, and an even smaller percentage had more than three treatment groups [4]. Although literature is available detailing the Bayesian methods that have been applied in the design and analysis of real clinical trials, there are very few reports on the implementation of Bayesian methods. The literature does not provide detailed information on how the studies were actually conducted, i.e., the computational approaches to the adaptation processes.

### Tools needed to conduct BAD

Bayesian trials depend on timely data entry for outcomes to inform the adaptation rules in real time [4] and this has presented additional challenges for investigators. While general computing tools have been developed in recent years to assist with some of the inherent computational demands of adaptation, specialized computer programs remain necessary to design and conduct a Bayesian study and to allow for replication by providing information on how the adaptation process was applied. In our review of published papers of BAD studies, it was nearly impossible to determine how the adaptive randomization was conducted. A multicenter breast cancer study (I-SPY 1) that integrated clinical, imaging, and genomic data to evaluate pathologic response, as well as their relationship and predictability based on tumor biomarkers, mentioned developing a web-based system called caINTEGRATOR as a common platform for data acquisition [6]. While the article provided a link to learn more about caINTEGRATOR, the webpage provided few details about the process or implementation of the adaptive randomization used. A clinical trial conduct (CTC) platform also was developed at the University of Texas MD Anderson Cancer Center to facilitate conducting Bayesian clinical trials [4]. Details about the functionality of the CTC platform used in the Biomarker-integrated Approaches of Targeted Therapy for Lung Cancer Elimination (BATTLE) trial, however, were limited to describing that after the patient’s eligibility criteria are entered by a nurse coordinator, the information is passed to an R script through web services, which performs Bayesian computations to determine the randomization probability and randomizes patients to eligible treatments accordingly [4]. Similarly, a Bayesian adaptive design for lung cancer therapy [7] provided few details.

An adaptive trial requires a system to manage random assignment to treatment arms. When the trial is multisite, a centralized randomization table is needed for use across all sites. With Bayesian methods, randomization is adapted at each interim analysis and it is, therefore, necessary to ensure that all sites begin using the updated allocation table at the same time [8] to uniformly support data acquisition. Integrating electronic data systems/capture and computational software clearly facilitates implementing and adopting Bayesian methods in clinical trials. For multisite studies, web-based applications are especially valuable primary tools to capture the data and provide for timely data entry and analysis. Web-based services also provide additional benefits such as exchanging information between the database module and the computing module [4].

This manuscript is the first to detail the technical process used to evaluate a multisite comparative effectiveness trial using adaptive randomization. An important opportunity for the application of Bayesian trials is in comparative effectiveness trials. The specific case study presented in this paper can be used as a model for conducting future clinical trials using a combination of statistical software and a web-based application. In this paper we describe how computational software programs were used for conducting a Bayesian adaptive clinical trial. The programs are used for generating new randomization tables that can be integrated in data capture tools in one comparative effectiveness trial.

## Methods

Our illustrative trial, Patient Assisted Intervention for Neuropathy*:* Comparison of Treatment in Real Life Situations (PAIN-CONTRoLS) is a multisite Bayesian prospective adaptive randomization trial with four arms that combines safety and efficacy. This ambitious trial was sorely needed, but it presented many logistical challenges. Because there have been no previous trials of drugs to treat pain in patients with cryptogenic sensory polyneuropathy (CSPN) [9], there not only has been no information to guide physicians treating CSPN patients, but insurance carriers often reject authorizing prescriptions for some drugs commonly used for other neuropathies. Therefore, we proposed conducting this comparative effectiveness prospective study using drugs commonly prescribed by physicians when caring for CSPN patients.

For illustrative purposes some brief additional information about our study is presented here. Cryptogenic sensory polyneuropathy (CSPN), also known as idiopathic polyneuropathy, is the diagnosis assigned when known causes of neuropathy have been excluded [9]. Seventy to eighty percent of CSPN patients present with pain ([9] and [10]). The trial goal is to identify which drug is most effective in reducing pain with the fewest side effects. Pain [11] at 12 weeks after study enrollment is the primary end point for our study. Study participants are randomized to one of four drugs and pain was measured at 4 and 8 weeks, in addition to our 12-week primary end point. Each participant is rated at each measurement time as either staying on the drug or quitting the drug due to lack of efficacy or adverse side effects (i.e., “quit” is not good). If the patient was able to remain on the drug, it was determined whether the drug was considered efficacious or not. Efficacy is defined as a 50 % or more reduction in the Likert pain scale from baseline to the follow-up visit. We estimated a need for 40 sites, with each site enrolling an average of 10 patients for a total sample size of 400. All of the sites are located in the USA except one located in Canada. We will be utilizing response-adaptive randomization based on the 4-, 8-, and 12-week patient outcomes. The adaptations are to occur after 80 patients have been enrolled and then every 13 weeks after that. For this study, a central Institutional Review Board (IRB) of record is the Human Subjects Committee and the ethics committee is the Human Research Protection Program, both at University of Kansas Medical Center. The IRB # is STUDY00001500. The study was first approved by the IRB on 24 September 2014.

### Bayesian Adaptive Design

The BAD for this study is driven entirely by the 12-week response (quit or not; and if still on the drug, efficacious or not). We label the response for patient *i* at weeks 4, 8, and 12 as vectors of length three, **Y**_**i**,**4**_, **Y**_**i**,**8**_, and **Y**_**i**,**12**,_ respectively, with each component of the three-dimensional vector representing the follow-up response for that patient. The sample size and the randomization ratio to each arm depend on the accumulating information in the trial.

First there is an initial Burn-in phase. For our study this was defined as equally randomizing 80 participants to the four arms (20 per arm.) Each participant must complete and sign a study consent form and upon randomization to the treatment drug, must then sign a second, drug-specific consent form. If the patient refuses to sign either, they will not be considered enrolled in the study. After the 80th participant is enrolled, any longitudinal data that is collected up to that point will be used to drive the first adaptive randomization. The Adaptive randomization phase, where adaptations occur every 13 weeks, comes after the Burn-in phase. In this phase, a vector of probabilities, ***q*** = (*q*_*1*_, *q*_*2*_, *q*_*3*_, *q*_*4*_), is created from an analysis of the current longitudinal data. From this point forward patients are allocated according to this, and subsequent, response-adaptive randomization vectors. At each update during the Adaptive randomization phase a decision is made on whether or not a best drug has been identified (i.e., the posterior probability of the *most effective drug* is at least 0.925.) If so, and if this occurs before enrolling all patients initially planned for, the trial is stopped for early success. The trial will be considered a success if either of the following occurs:

1. Early success: if at any interim analysis the most likely arm has at least a 0.925 posterior probability of being the best arm
2. End-of-trial success: if at the conclusion of participation by 400 patients, the most likely arm has at least a 0.925 posterior probability of being the best arm

While there may not be a single clear best arm, any arm with a posterior probability of no more than 0.01 will be designated a “loser” and no more patients will be randomized to that arm. This value was selected based on data from simulations that suggested a higher posterior probability value would lead to an unacceptable “loser” false discovery rate.

The study team investigated several operating characteristics including power, trial size, patient allocation across arms, and trial duration. From these simulations, it was learned that the trial has a Type I error rate of approximately 5 %. In the likely scenario of one best arm, the trial has 94 % power with an average sample size of 266, well below the 400, with over half of those patients receiving the best treatment. The design for this trial also allows for the declaration of loser arms if no true winner is present.

### End point and longitudinal statistical modeling

The response for patient *i*, at the 12-week visit is a three-dimensional vector **Y**_**i**,**12**_. The model has a multinomial distribution **Y**_**i**,**12**_ which approximates to *Multinomial*(θ**a**_**i**_), where *a*_*i*_ is the treatment arm for patient *i*. The response to pain medication (rates of quit and efficacy) at 12 weeks for arm *a* is a three-dimensional vector θ_**a**_ (quit; not quit and efficacious; not quit and not efficacious.) Uniform priors are provided (θ_**a**_) approximating to Dirichlet (1/3,1/3,1/3) [12]. To measure the utility of an arm the components of θ_**a**_ are combined to obtain a utility, *U*_*a*_, for the *a*^th^ drug. The formula is:

$$ {U}_a=0.75{\uptheta}_{a, 2}+\left(1-{\uptheta}_{a, 1}\right), $$

where θ_*a*,*1*_ and θ_*a*,*2*_ represent the quit rate and efficacy rate respectively for drug *a*.

This utility function was chosen after discussion with clinical experts regarding the relative utility of quit and efficacy, a process described in Gajewski et al. [12].

At each interim analysis some patients will have complete information on their 12-week observation, **Y**_**i**,**12**_. These patients may also have their interim values of response to pain medication observed, **Y**_**i**,**4**_ and **Y**_**i**,**8**_. There also will be patients with interim responses, but no 12-week value, and patients with no responses. We use information from patients with incomplete information to the extent that the interim values from those with complete data are predictive of the final 12-week values.

A conditional multinomial model is created for the prediction between each interim time period response value and the 12-week values. A separate version of the model is used for each experimental arm and each time period. The multinomial model is used only on patients who stay on medication, as anytime a patient quits a medication subsequent observations are also “quit”’ The priors used for these models are as follows: we let p21, p22, and p23, the 12-week probabilities of quit, not quit efficacy, and not quit no efficacy, be conditional on a patient showing early efficacy at 4 or 8 weeks. For these we use a Dirichlet prior, (p21, p22, p23) which approximates to Dirichlet (1, 7, 2), representing a priori mean rates of 10 %, 70 %, and 20 % at 12 weeks for early efficacy. These priors were elicited from clinical experts. For a patient who shows no efficacy early, the final prior probabilities are (p31, p32, p33) which approximates to Dirichlet (1, 2, 7), representing a priori mean rates of 10 %, 20 %, and 70 % at 12 weeks for patients showing early no efficacy. These are fairly “weakly informative,” each having a prior sample size equivalent to 10 patients.

### Bayesian quantities

The following Bayesian quantities, calculated at each interim analysis, are used in the adaptive design. The posterior probability that each arm, *a* = 1,2,3,4, is the maximally effective arm, *P*_*a*_^max^, is calculated from the joint posterior distribution. The arm with the largest *P*_*a*_^max^ is labeled the *most likely maximum effective drug*, as determined by the posterior utility function (i.e., *P*_*a*_^max^ is a function of *U*_*a*_). The posterior mean and variance for each utility also is calculated. We label *V*(*U*_*a*_) as the posterior variance of the parameter *U*_*a*_.

### Adaptive randomization

As previously identified, during the initial Burn-in phase (with 80 patients in our trial) allocation is set at 1:1:1:1 for arms 1, 2, 3, and 4, respectively. After the Burn-in phase, adaptive allocation randomization is used in which the allocation probabilities are updated quarterly to favor those drugs most likely to be the best. After exploring the operating characteristics for monthly, bi-monthly, and quarterly adaptations, quarterly adaptations were decided upon for a couple reasons. The operating characteristics did not significantly diminish when performing quarterly adaptations, but the time and resource burden for the study team were drastically reduced. The randomization vector of probabilities is created by selecting a vector based on the posterior distribution of the utility function for each arm.

The posterior variance of *U*_*a*_ for each drug, and the posterior probability that each drug is the best arm, is used for the specification of the randomization vector. Let the number of patients enrolled in arm *a* be *n*_*a*_. The goal of adaptive randomization is to allocate more patients to the arms most likely to be the best, as well as to learn about which arm is best. A *variance component*, *V*_*a*_, as well as the probability drug *a* is the *maximally effective arm*, *P*_*a*_^max^, are, therefore, constructed for each arm. The information for arms *a* = 1,2,3,4 is:

$$ {I}_a=\sqrt{P_a^{max}V\left({U}_a\right)/\left({n}_a+1\right)}. $$

The randomization probabilities for each of these drugs will be proportional to *I*_*a*_. The randomization vector, ***q***, is set as *q*_*a*_ = *I*_*a*_/∑*I*_*a*_. The information measure was chosen because it randomizes more patients to the arm with higher standard error if all posterior probabilities that the arm is the best are the same. Sole allocation based on probability does not have this property.

**Fig. 1:**
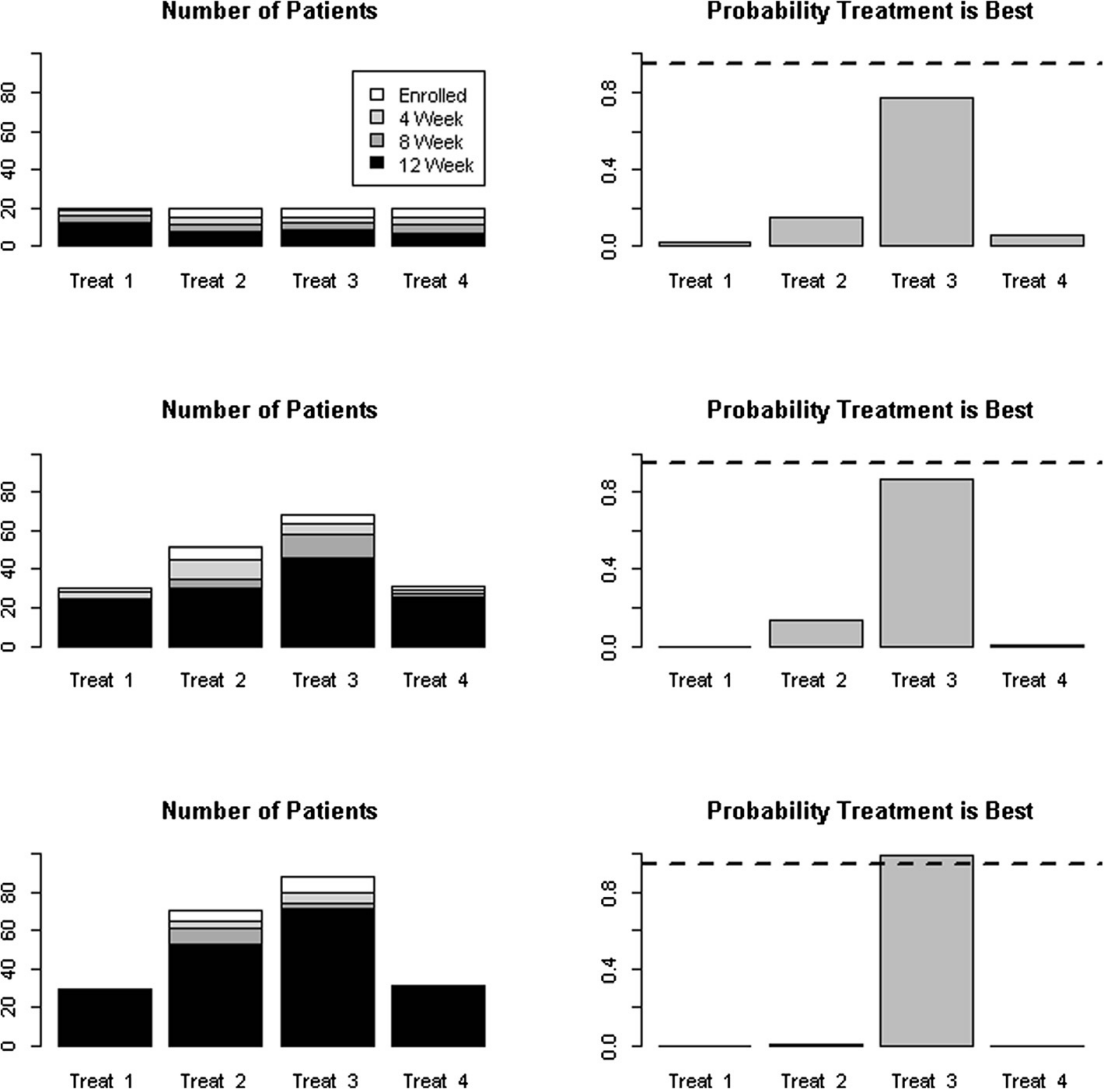
An example of first, second, and third interim analysis

Three example interim analyses are shown in Fig. 1. In the first interim analysis a new adaptive randomization is calculated after the initial equal allocation to each group. Some of the patients have no data while others have 4-, 8-, or 12-week data. The adaptive randomization uses all patient data with at least 4 weeks of data. As seen in Fig. 1, the first test interim analysis shows Treatment 3 trending toward the “best” but not quite at the predefined success criterion. The second interim analysis shown has many more mock patients and, consistent with the adaptive randomization approach, more are randomized to Treatment 3 but the study has not been stopped for success. The third interim analysis shown indicates “success,” and Treatment 3 is deemed the best. Note that the bottom-most histogram reflects that no patients were enrolled in Treatments 1 and 4 after the second interim analysis because the posterior probabilities for those arms were 0.01 or less and, thus, they were deemed “losers.”

**Fig. 2:**
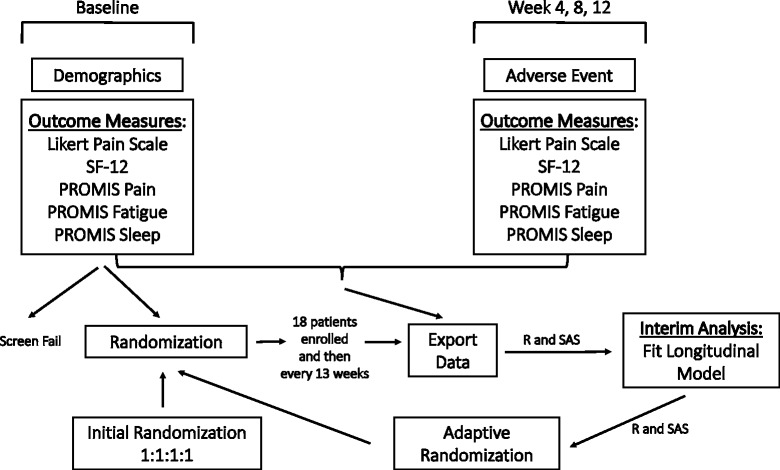
Overall schematic of the trial’s conduction

### Trial computational tools (REDCap™, SAS®, R)

For our study we developed an in-house computation software program built in REDCap™ [13], SAS® [14], and R [15]. This platform provides a smooth transition between randomization sequences at each adaptation during the trial (Fig. 2), without interference to any of the multiple sites. REDCap (http://project-redcap.org/) [13] is a popular web application used for capturing research study data, and while a basic randomization module exists within REDCap, a more novel model was required for our longitudinal, multisite BAD. SAS® and R were used to clean the data, perform the analyses, and generate the new allocation ratios and randomization table. Our enhancements work in conjunction with REDCap to accomplish response-adaptive randomization. Our design is just one example, but we believe that researchers using BADs would benefit from adopting, and modifying if needed, the web-based centralized system we developed and present here.

Some logistical challenges arise in multisite trials when the randomization table needs to be updated at every interim analysis. Our trial is conducted at 40 sites across the country and each site began, or will begin, enrollment at different times. It was decided that an Internet-based randomization process would be most effective— especially since clinicians needed to provide the patient with a prescription for their treatment drug at the patient’s clinic visit. An Internet-based randomization process also enables all sites to use one master allocation table and ensures that only one allocation table needs updating by the statistician at each response-adaptive randomization. Another goal was to ensure that none of the sites would be affected by any lag in the study process when an adaptation in the randomization sequence occurred, and having one master Internet-based allocation table has allowed us to achieve that goal.

Using one electronic data capture tool database, REDCap [13], across all sites also helped to reduce some of the logistical challenges. It was determined that utilizing one stand along system, such as REDCap, that allows the researchers across all the sites to collect the data in a timely manner and would allow real-time randomization, was most efficient and effective for this trial. The lead investigator’s institution, the University of Kansas Medical Center, hosts the data warehouse and portal for data collection, storage, and management from all sites. The REDCap tool contains eight forms to capture all data needed. Within REDCap, each site is assigned its own data access group to ensure that team members from each site had access to only the patients from their site.

### Conducting a BAD trial using REDCap

More than 157,000 projects created by more than 216,000 users from nearly 1400 institutions in 88 countries have used REDCap and published over 1365 articles since 2008 [16]. Of these, just over 60 studies are randomized trials, and only one article reported using a BAD [16]. Not surprisingly, details about how adaptive randomization was implemented in the REDCap system were not reported. By describing how the adaptive randomization was implemented for our study using this widely available program, we hope to begin to address this gap in the literature.

The front end of the REDCap application is built on a server-side scripting language designed for web development (PHP) and the backend of the application resides on a MySQL database. REDCap has a default built-in module for randomization. The allocation table for our BAD trial is a random combination of four different numbers (i.e., 1, 2, 3, and 4), each corresponding to a different study drug. It should be noted that although this trial chose not to stratify the randomization by site, due to a small expected number of patients to be enrolled across all the sites, the REDCap application has the capabilities to include a stratification variable in the randomization table. This would function well for traditional, stratified randomization trials and for stratified response-adaptive randomization trials.

**Fig. 3:**
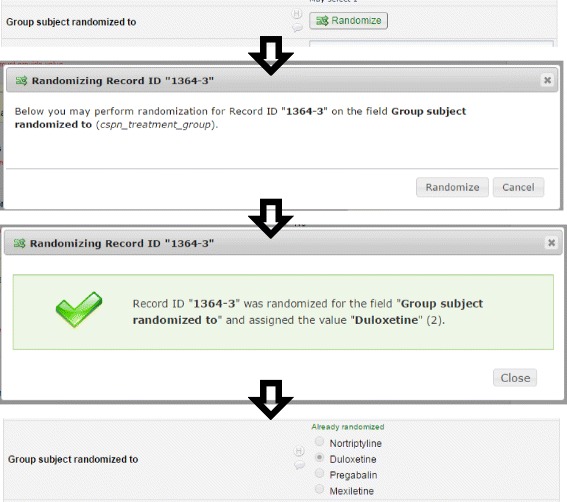
Screen shots from the randomization procedure within REDCap

The initial allocation table is uploaded and attached to the study through the built-in module within REDCap. Once basic demographics have been entered for a patient, a banner is displayed to the research site coordinator indicating whether or not the patient is eligible for the study. If eligible, the site coordinator clicks the randomization button on the study form and the next available slot on the allocation table is assigned to that patient Fig. 3. Twenty extra slots were added to the allocation table in case the 81st patient needed to be randomized while we prepared the next adapted allocation table. Once we assign 80 of these 100 slots, the data from REDCap is extracted and analyzed in SAS and then R is used to generate the new allocation table. Any remaining unassigned slots in the first randomization table are deleted and the new table is appended to the existing table. This process is repeated every 13 weeks using available data to repeat the adaptive randomization process.

### Another trial tool – eResearch

Another option available to investigators is eResearch, a secure, web-based clinical trial management tool that can be used to maintain all the study information and research patient information [17]. The front end of eResearch is built on Java server pages (JSP) and the backend (i.e., the database) resides on Oracle 11G. This application includes all the same features available in REDCap, including the capability of performing fixed or adaptive randomization. The only difference is that eResearch focuses more on clinical usage, as it is primarily a clinical trial management tool, while REDCap is strictly a data capture tool. Based upon study preferences and priorities, investigators may use whichever tool best suits their needs.

Another major challenge that comes with using an electronic data capture system is the possibility that a computer, or Internet access, may not be available in the clinic during the patient’s visit. While a reliable paper backup of the study forms for data capture is available for such an event at each site, research staff at the lead investigator’s institution must be available (by phone) in case the randomization module would also be unavailable. This ensures that the clinician would still have the ability to randomize the patient during the clinic visit and provide a prescription at that time.

## Results

As each adaptation in the allocation table approaches we are ensuring that the follow-up data for the study patients is being entered into the electronic database in a timely matter. Most sites are performing direct data entry and sites are being queried if a patient’s follow-up visit data is not in at the scheduled visit time. Data cleaning is also being performed every few weeks to verify the data quality. Any discrepancies or missing data values are being directed to the corresponding site coordinator for correction or clarification. The site coordinators will be correcting any patient data directly in the REDCap database. This ensures that the next time the data is extracted it will be correct and as up-to-date as possible. REDCap has an excellent logging tool that allows us to keep track of any changes that were made to the data, the user who made the changes, and when those changes were made. We wanted to ensure that all of the data was error free and complete as we approach the end of each portion of enrollment. For the adaptation, it is important for us to be able to use all of the longitudinal patient data that has been collected to inform our new allocation table. Larger studies may want to consider utilizing automated email messages to clinical staff to remind them of the patient’s follow-up schedule.

Prior to the first adaptation, we were working on developing an automated process, which would enable us to: (1) extract the data, (2) run the data cleaning process, (3) determine if a patient was categorized as quit, not quit and efficacious, or not quit and not efficacious for each visit, (4) generate the new randomization sequence needed for the next cohort of patients; and (5) append that new sequence to the existing allocation table seamlessly, without disturbance to any of the sites that were currently enrolling patients. The 80th patient was randomized to a study drug on 3 December 2015. The data was extracted from REDCap that evening. The extracted data was cleaned, and analyzed using SAS 9.4. For each time point the patients had completed, we determined if they were categorized as quit, not quit and efficacious, or not quit and not efficacious for each visit. A file was created from SAS containing the longitudinal data for each patient. The longitudinal model algorithm was programmed in R. The R program used the file that was created in SAS to calculate the posterior probabilities of the treatment arms (interim analysis) and to generate the new allocation table using the SAMPLE function in R. The new table was then appended to the existing randomization table within REDCap on the afternoon of 4 December. In total, it took less than 24 hours from when the 80th patient was randomized to upload and have the new randomization table that utilized the new allocation probabilities ready for the next patient. Finally, after a site had randomized another patient, it was confirmed that the patient was randomized to the treatment drug that was found in the first spot of the new randomization table.

There are now 154 total patients enrolled on the study, with 33 sites currently enrolling. We have performed an additional response-adaptive randomization, which occurred in March, and the same process was utilized from the first adaption. It went very smoothly and we were able to extract the data and append the new randomization table with the updated allocation ratios to the REDCap database within two business days.

## Discussion

Due to this trial being completely unblinded, in general, physician bias could be an issue. However, the physician was left unblinded for two reasons. First, the sponsor’s (PCORI) goal is to emulate real clinical practice (i.e., pragmatic) and wanted this to be unblinded. Second, the physicians at each site are enrolling a small number of patients and are enrolling very slowly. We did not expect them to develop any bias to prescribing one of the four treatment groups. It should be noted that after each response-adaptive randomization, a Data Safety Monitoring Board (DSMB), along with the enrolling physicians, receive a report that details the adaptation. We have given the treatment drugs generic labels (A, B, C, D) so as to blind the DSMB and enrolling physicians from which drugs are performing better or worse than the others.

While we have pointed out many of the benefits of response-adaptive randomization, there are some drawbacks that should be mentioned. Meurer et al. described some barriers to the adoption of adaptive clinical trial design as “increased complexity during both study design and trial conduct,” and “questions about the receptiveness of funding agencies and peer reviewers” [18]. Some of the complexity during trial conduct, specifically the changes in randomization ratios that need to be periodically implemented, has become more available due to computerized central randomization. The innovative process we describe in our paper is a prime example of how we were able to achieve this.

## Conclusions

The Bayesian Adaptive Design (BAD) was chosen with efficiency in mind. Using adaptive randomization not only allows for substantially smaller sample sizes, but also provides better conclusions about what treatments are the most effective, because it lets us make changes to our approach or stop the study early if we find strong results before the scheduled end of the study. While the study is still enrolling, the first adaptation in the randomization provided drastic changes to the initial 1:1:1:1 allocation ratio.

REDCap is a popular tool used by many for randomized clinical trials. As more Bayesian clinical trials are being conducted in practice, additional information about how this program can be used for BAD trials is needed. In this paper we detailed how an adaptive randomization process was developed for use with REDCap as an example for conducting a BAD comparative effectiveness trial where data capture and randomization are accomplished in real time. Our specific case study should serve as a model for future clinical trials using a combination of statistical software and web-based applications.

## Abbreviations

CSPN: Cryptogenic sensory polyneuropathy
CTSA: Clinical and Translational Science Awards
NCATS: National Center for Advancing Translational Sciences
NIH: National Institute of Health
PAIN-CONTRoLS: Patient Assisted Intervention for Neuropathy: Comparison of Treatment in Real Life Situations
PCORI: Patient Centered Outcomes Research Institute
